# Accumulation of N-Isopropyl-p(123I)iodoamphetamine on Single-Photon Emission Computed Tomography Between Immunosuppressed and Non-immunosuppressed Patients With Primary Central Nervous System Lymphoma

**DOI:** 10.7759/cureus.82763

**Published:** 2025-04-22

**Authors:** Yuki Sakaeyama, Yutaka Fuchinoue, Chie Matsuura, Masaaki Nemoto, Ryo Matsuzaki, Shuhei Kubota, Mitsuyoshi Abe, Sayaka Terazono, Nobuo Sugo

**Affiliations:** 1 Department of Neurosurgery (Omori), Faculty of Medicine, Toho University, Tokyo, JPN; 2 Department of Neurosurgery (Sakura), Faculty of Medicine, Toho University, Chiba, JPN

**Keywords:** immunosuppressed, n-isopropyl-p-[123i]-iodoamphetamine single-photon emission computed tomography, non-immunosuppressed, primary central nervous system lymphoma (pcnsl), tumor immune microenvironment

## Abstract

Introduction

Primary central nervous system lymphoma (PCNSL) is a rare type of malignant tumor. Due to the rapidly progressive nature of PCNSL, early diagnosis is important, and imaging plays a key role in this process. In particular, N-isopropyl-p(123I)iodoamphetamine (^123^I-IMP) single-photon emission computed tomography (SPECT) is useful for PCNSL diagnosis because it shows a high accumulation, which distinguishes the lesion from other brain tumors. Recently, PCNSL has been increasingly observed in immunosuppressed patients, including those who have undergone organ transplantation and those who are receiving treatment for acquired immunodeficiency syndrome. These conditions may alter the tumor microenvironment, thereby potentially influencing ^123^I-IMP accumulation. The current study aimed to validate how immunosuppression affects the diagnostic imaging of PCNSL using ^123^I-IMP SPECT.

Materials and methods

This study included 12 patients diagnosed with PCNSL based on surgical specimens, all of whom underwent early and delayed ^123^I-IMP SPECT imaging. The patients were divided into the immunosuppressed and non-immunosuppressed groups. The immunosuppressed group consisted of three patients who received steroids or immunosuppressants after kidney transplantation. Seven tumors from the three patients in the immunosuppressed group and nine tumors from the nine patients in the non-immunosuppressed group were compared. Early and delayed SPECT images were obtained, and the regions of interest were defined by fusing SPECT with magnetic resonance imaging. The tumor-to-cerebellum (T/C) ratio was calculated, and statistical analysis was performed using the Mann-Whitney U test.

Results

The immunosuppressed group had a significantly lower T/C ratio on the early ^123^I-IMP SPECT images than the non-immunosuppressed group (0.57 ± 0.14 vs 0.83 ± 0.14, p < 0.01). The immunosuppressed group also had a significantly lower accumulation on the delayed images than the non-immunosuppressed group (0.84 ± 0.16 vs. 1.22 ± 0.10, p < 0.001). The T/C ratio showed a statistically significant increase from the early to delayed images in both the immunosuppressed and non-immunosuppressed groups (p < 0.01, p < 0.001).

Conclusions

The immunosuppressed group had a significantly lower ^123^I-IMP accumulation in the early and delayed images than the non-immunosuppressed group. Based on this finding, an immunosuppressed state strongly influences the ^123^I-IMP uptake in PCNSL. Thus, the presence or absence of an immunosuppressed state should be considered when diagnosing PCNSL using ^123^I-IMP SPECT.

## Introduction

Primary central nervous system lymphoma (PCNSL) is a rare type of malignant tumor that develops in the brain, spinal cord, or eyes, with an incidence of 0.44 cases per 100,000 people [1]. PCNSL progresses rapidly, and early treatment significantly affects the prognosis of patients. Therefore, early diagnosis is important, and imaging plays a key role in this process. Single-photon emission computed tomography (SPECT) using N-isopropyl-p(123I)iodoamphetamine (^123^I-IMP) is essential in the diagnostic imaging of PCNSL. ^123^I-IMP is a useful differential diagnostic tool for PCNSL because it shows a high accumulation. Meanwhile, other brain tumors often present with a low accumulation [2-5]. In recent years, PCNSL has been increasingly observed not only in patients without underlying conditions but also in those with an immunosuppressed state (such as those who have undergone organ transplantation, those receiving immunosuppressive therapy, and those with acquired immunodeficiency syndrome) [6]. Under these immunosuppressive conditions, angiogenesis, immune cell infiltration, and the metabolic activity within the tumor microenvironment are affected [7,8]. Therefore, the ^123^I-IMP uptake can possibly be affected in PCNSL under immunosuppressive conditions; however, this notion remains to be elucidated. The current study aimed to validate how immunosuppression affects the diagnostic imaging of PCNSL using ^123^I-IMP SPECT.

## Materials and methods

This study included 12 patients who were diagnosed pathologically with PCNSL based on surgical specimens between June 2012 and February 2025 and who underwent early and delayed ^123^I-IMP SPECT imaging. The patients were divided into the immunosuppressed and non-immunosuppressed groups. The immunosuppressed group included three patients: one who received steroids for brain edema and two who received immunosuppressants after kidney transplantation for IgA nephropathy.

**Figure 1:**
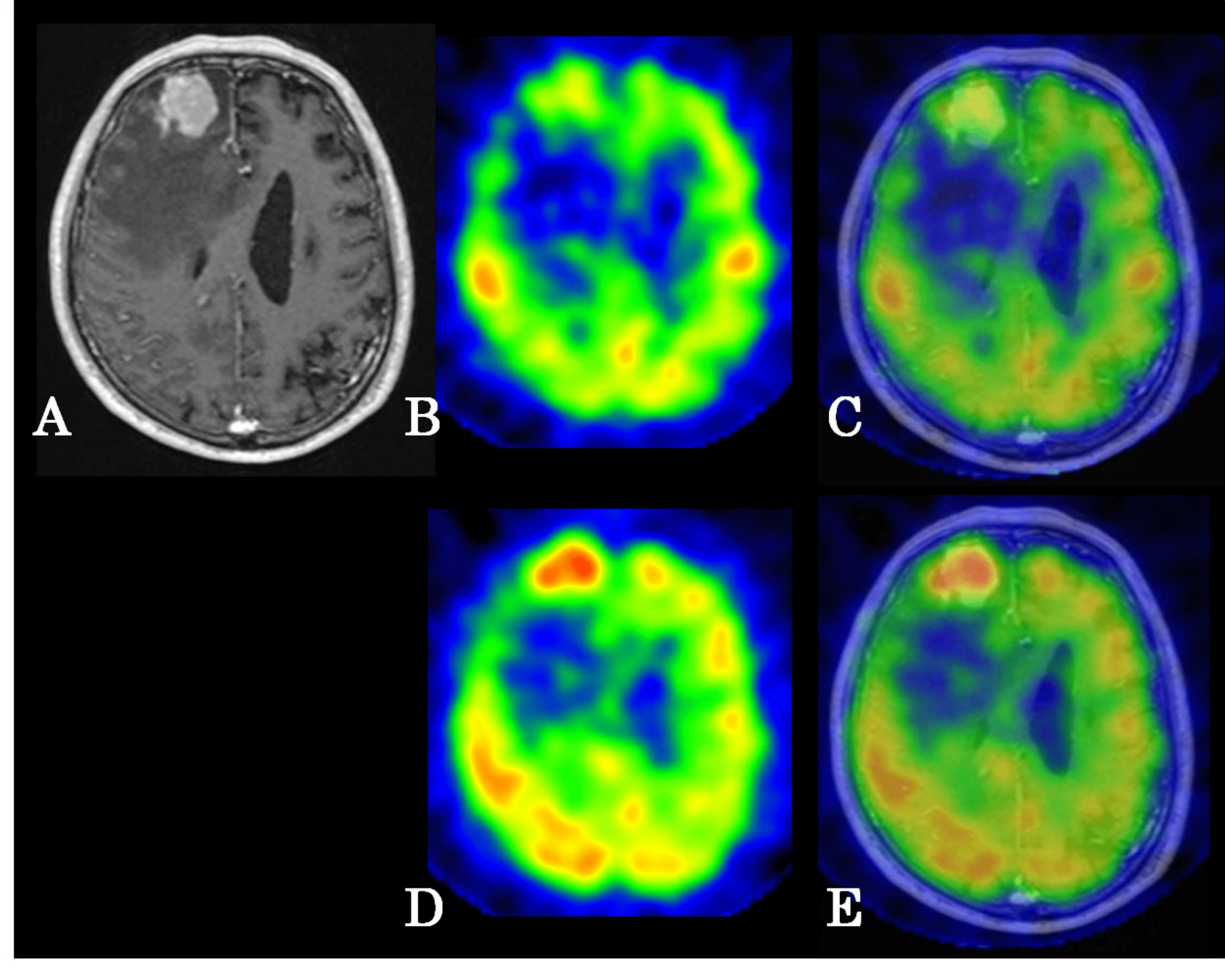
Representative case from the non-immunosuppressed group had a tumor in the right frontal lobe (A) Gadolinium-enhanced T1-weighted image, (B) early image of SPECT, (C) fusion image of gadolinium-enhanced T1-weighted imaging and early image of SPECT, (D) delayed image of SPECT, (E) fusion image of gadolinium-enhanced T1-weighted imaging and delayed image of SPECT. SPECT: Single-photon emission computed tomography

**Figure 2:**
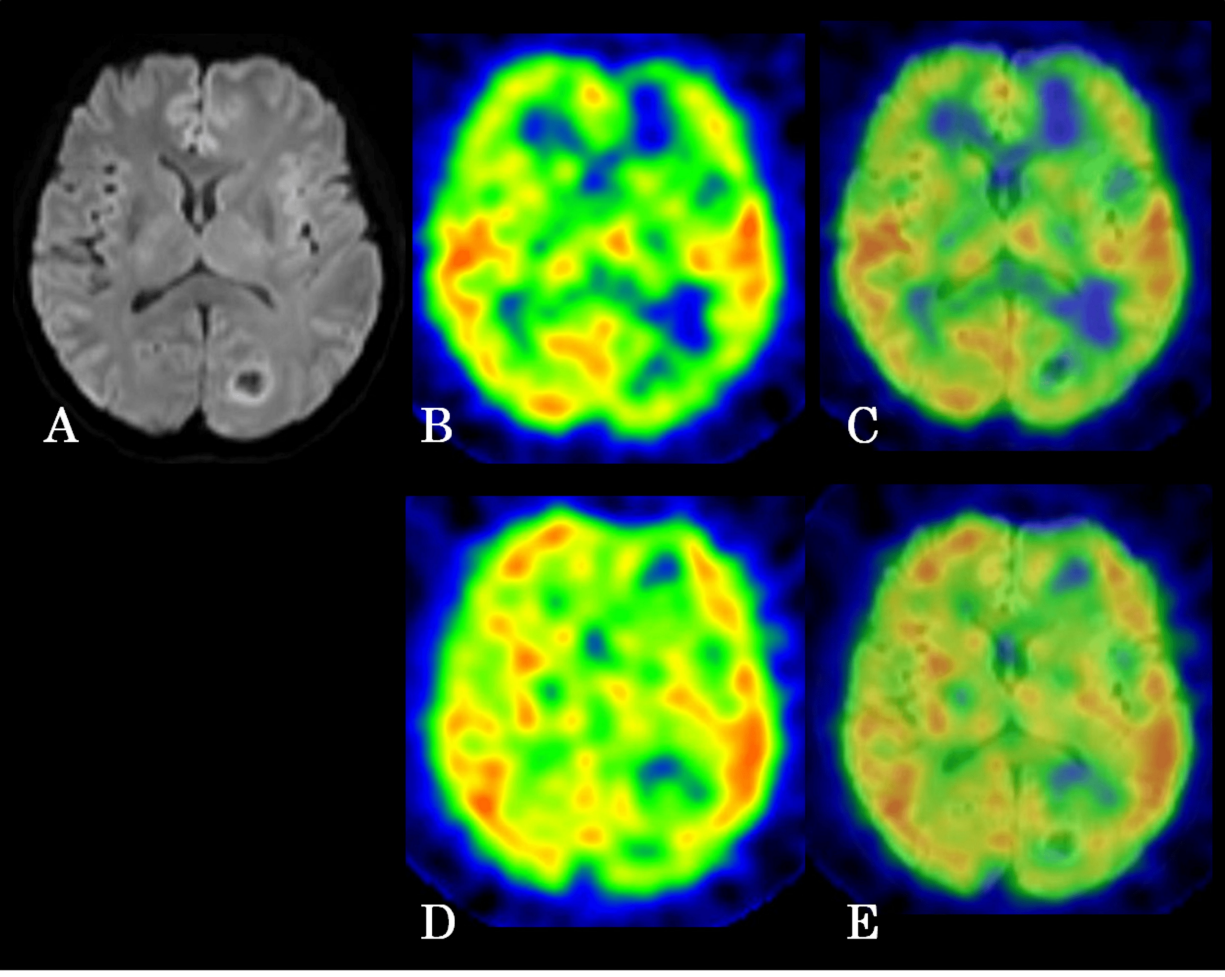
Representative case from the immunosuppressed group had a tumor in the left occipital lobe (A) Diffusion-weighted magnetic resonance image, (B) early image of SPECT, (C) fusion image of diffusion-weighted MRI and early image of SPECT, (D) delayed image of SPECT, (E) fusion image of diffusion-weighted MRI and delayed image of SPECT. SPECT: Single-photon emission computed tomography

Among the two kidney transplant recipients, one had three tumors in the ipsilateral cerebral hemisphere. The other had one tumor in the left hemisphere and two in the corpus callosum. Seven tumors from three patients in the immunosuppressed group and nine tumors from nine patients in the non-immunosuppressed group were compared using early and delayed ^123^I-IMP SPECT imaging. A three-detector SPECT system (GCA-9300R; Canon Medical Systems Co., Tochigi, Japan) was used. A fan-beam high-resolution collimator with a system resolution of 1 cm was also utilized. Data were collected under the following specifications: the acquisition mode was set to 25 min with a matrix size of 128 × 128, a sampling angle of 4°, and a single direction time of 50 s. The acquisition conditions included an energy window centered at 159 keV ± 10%, a turning radius of 132 mm, and a magnification factor of 1.0. During the procedure, the patients were placed in the supine position and were administered ^123^I-IMP (222 MBq) intravenously. Data collection started 15 min post-injection, and it was continued for 30 min. Early images were acquired 15-30 min post-injection. Meanwhile, delayed images were obtained after 4 h under similar conditions. For image reconstruction, the filtered back-projection method was applied using the Butterworth filter with a cutoff frequency of 0.6-0.9 cycles/pixel and an order of four. Collimator aperture correction was performed; however, absorption and scattering corrections were not applied. To accurately define the region of interest (ROI) corresponding to the tumor on SPECT, a fusion image with magnetic resonance imaging (MRI) was created using a three-dimensional image analysis system (SYNAPSE VINCENT; Fuji Film, Tokyo, Japan) that was designed for medical use. MRI was performed with a 1.5-T MRI scanner (Excelart Vantage MRT-2003/P3; Toshiba, Tokyo, Japan). Depending on the case, the fusion image with SPECT was generated using either a gadolinium-enhanced T1-weighted image or a diffusion-weighted image, whichever is best for visualizing the tumor. The ROI was then defined accordingly (Figures 1A-1E, 2A-2E).

The ROI was set in the cerebellum ipsilateral to the tumor on ^123^I-IMP SPECT. The tumor-to-cerebellum (T/C) ratio was calculated using the following formula: T/C ratio = (tumor accumulation (counts/pixel))/(accumulation in the normal ipsilateral cerebellum (counts/pixel)) [2]. The Mann-Whitney U test was used to compare the two groups. P values < 0.05 indicated statistically significant differences.

## Results

Table 1 shows the summary of the cases in the immunosuppressed and non-immunosuppressed groups.

**Table 1:** Summary of cases M: male, F: female

		Immunosuppressed group			Non-immunosuppressed group		
N		3			9		
Age		67.0 ± 11.8			71.3 ± 8.9		
Sex							
M		1			7		
F		2			2		
Number of tumors							
Single tumor		1			9		
Three tumors		2			0		
Total number of tumors		7			9		
Tumor location							
Frontal		2			6		
Temporal		1			0		
Parietal		1			0		
Occipital		1			0		
Basal ganglia		0			1		
Intraventricular		0			1		
Corpus callosum		2			1		

**Figure 3:**
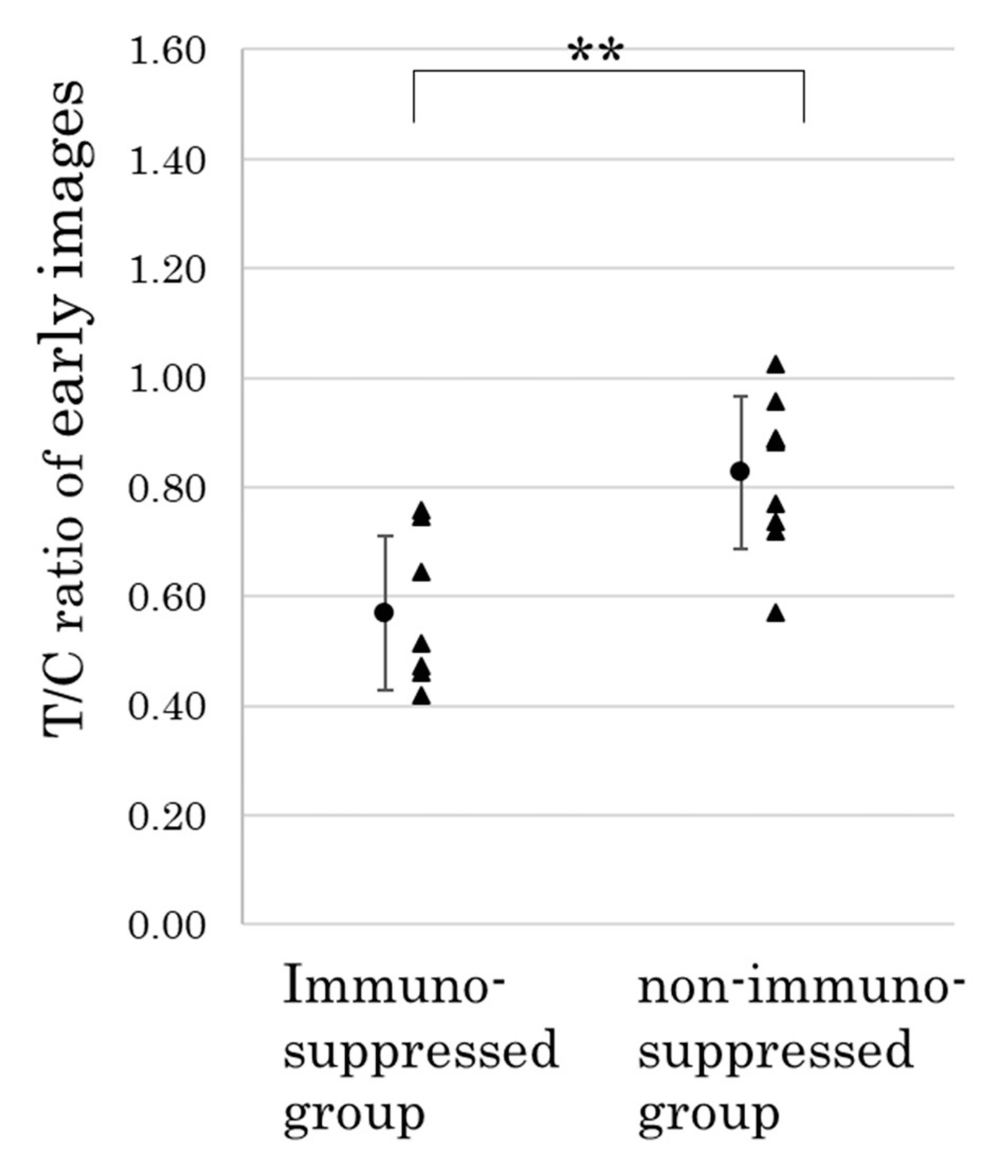
Tumor-to-cerebellum (T/C) ratio) in the ROI set on the cerebellum ipsilateral to the tumor in the early images of 123I-IMP SPECT ROI: region of interest, SPECT: Single-photon emission computed tomography

Diffuse large B-cell lymphoma was the pathological diagnosis of all the patients. The T/C ratios of eight tumors in the immunosuppressed group and nine tumors in the non-immunosuppressed group were compared using early ^123^I-IMP SPECT images. The immunosuppressed group had a significantly lower ^123^I-IMP accumulation than the non-immunosuppressed group (0.57 ± 0.14 vs. 0.83 ± 0.14) (p < 0.01, Figure 3).

**Figure 4:**
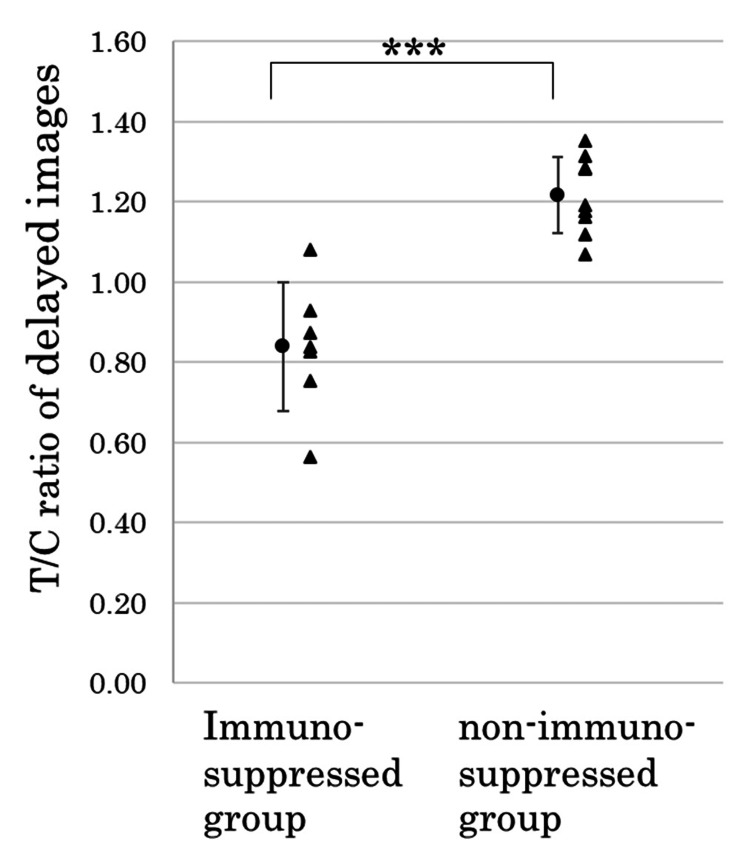
Tumor-to-cerebellum (T/C) ratio in the ROI set on the cerebellum ipsilateral to the tumor in the delayed images of 123I-IMP SPECT ROI: region of interest, SPECT: Single-photon emission computed tomography

In addition, the immunosuppressed group had a significantly lower ^123^I-IMP accumulation on the delayed images than the non-immunosuppressed group (0.84 ± 0.16 vs 1.22 ± 0.10) (p < 0.001, Figure 4).

The T/C ratio showed a statistically significant increase from the early to delayed images in the immunosuppressed and non-immunosuppressed groups (p < 0.01, p < 0.001).

## Discussion

Several studies have shown that a high accumulation of ^123^I-IMP SPECT is observed in PCNSL [2-5,9]. In 1989, Ohkawa et al. reported that a patient with PCNSL exhibited a high accumulation in both the early and delayed images of ^123^I-IMP SPECT. Thus, this specific imaging finding can be used for the diagnosis of PCNSL [9]. Subsequently, Akiyama et al. performed ^123^I-IMP SPECT in 12 cases of PCNSL and 84 cases of other brain tumors. They found that delayed images showed a high accumulation at the tumor site in patients with PCNSL. Thus, ^123^I-IMP SPECT is useful for PCNSL diagnosis [2]. Other studies have also revealed that ^123^I-IMP has a higher retention rate in PCNSL than in glioblastoma and meningioma. Thus, it is a characteristic finding of PCNSL and capturing early and delayed images with ^123^I-IMP SPECT is important for PCNSL diagnosis [3-5]. In the current study, regardless of whether the patient was in the immunosuppressed or non-immunosuppressed group, the delayed images had a significantly higher ^123^I-IMP accumulation than the early images. Hence, similar to the results of previous reports, this finding indicates that imaging is essential for PCNSL diagnosis.

The mechanism of ^123^I-IMP accumulation in brain tumors may differ between the early and delayed images. Brain tumors with a high accumulation on early ^123^I-IMP images are often meningiomas, which have an abundant blood flow. Further, the mechanism behind this is the intravascular retention of ^123^I-IMP [10,11]. In contrast, the mechanism of ^123^I-IMP accumulation on delayed images involves the interaction with nonspecific amine-binding sites in the brain [12]. Previous studies have reported that ^123^I-IMP shows a high accumulation on delayed images, not only in PCNSL but also in conditions such as cellular blue nevus and bronchial carcinoid tumors [13,14]. These tumors express abundant amine-binding receptors, which are associated with the production of melanin or monoamines, and ^123^I-IMP is retained throughout the delayed phase by binding to these receptors [3]. A novel finding of this study was that the immunosuppressed group had a significantly lower ^123^I-IMP accumulation on both the early and delayed images than the non-immunosuppressed group. In PCNSL under immunosuppressed conditions, immunosuppressive cells such as M2 macrophages and regulatory T cells accumulate within the tumor microenvironment and, thus, suppress the immune response. This reduces the infiltration of cytotoxic T cells and natural killer cells, which are immune cells that attack cancer cells, thereby weakening the immune response. A decrease in immune cell activity reduces the ^123^I-IMP uptake, which accumulates based on blood flow and cell activity [15,16]. Moreover, the suppression of angiogenesis by M2 macrophages in an immunosuppressive environment could also be a factor. The vascular function of the immunosuppressed groups is impaired, thereby reducing blood flow to the tumor, which may result in insufficient ^123^I-IMP accumulation [17]. PCNSL responds well to steroid therapy [8]. The size of PCNSL decreases with steroid administration due to the following reasons: the immunosuppressive effect of steroids, induction of apoptosis in tumor cells, and infiltration of macrophages into the tumor, which enhances the inflammatory response [8]. These mechanisms can reduce tumor size and decrease ^123^I-IMP accumulation. Based on the results of this study, when using ^123^I-IMP SPECT as an auxiliary imaging diagnostic tool for PCNSL, it is necessary to consider that ^123^I-IMP does not necessarily show a high accumulation in tumors under immunosuppressed conditions.

Limitation

The limitation of this study is that it had a small number of cases. However, PCNSL is a rare disease, and multicenter collaborative studies are anticipated in the future.

## Conclusions

The ^123^I-IMP accumulation increased from the early to delayed images in both the immunosuppressed and non-immunosuppressed groups with PCNSL. Therefore, this finding is specific to PCNSL. However, the immunosuppressed group had a significantly lower ^123^I-IMP accumulation on both the early and delayed images than the non-immunosuppressed group. This finding suggests that an immunosuppressed state significantly affects the ^123^I-IMP uptake in PCNSL. Therefore, the presence or absence of an immunosuppressed state should be considered when diagnosing PCNSL using ^123^I-IMP SPECT.
